# Supplementary material for: A high-resolution mRNA expression time course of embryonic development in zebrafish
Source: eLife. 2017 Nov 16;6:e30860. doi: 10.7554/eLife.30860 (PMC5690287; doi:10.7554/eLife.30860)
Supplement: Supplementary file 6. [file elife-30860-supp6.zip › biolayout-clusters-files/Cluster119-genes.html]

Cluster119


# Cluster119: Genes

| Ensembl ID | Gene Name | Chr | Start | End | Biotype |
| --- | --- | --- | --- | --- | --- |
| ENSDARG00000077785 | atf5b | 15 | 17929973 | 17934068 | protein\_coding |
| ENSDARG00000036102 | ctdsp2 | 23 | 24742494 | 24755703 | protein\_coding |
| ENSDARG00000071558 | fblim1 | 22 | 185077 | 201869 | protein\_coding |
| ENSDARG00000087697 | lpl | 22 | 15598222 | 15603932 | protein\_coding |
| ENSDARG00000020328 | plekha6 | 11 | 23089972 | 23316539 | protein\_coding |
| ENSDARG00000003008 | prkcea | 12 | 25549120 | 25678872 | protein\_coding |
| ENSDARG00000035970 | ptprn2 | 7 | 39957345 | 40228369 | protein\_coding |
| ENSDARG00000077424 | rassf10a | 25 | 16343046 | 16344350 | protein\_coding |
| ENSDARG00000097845 | si:ch211-105j21.9 | 6 | 49062424 | 49064432 | protein\_coding |
| ENSDARG00000095965 | si:ch211-268b13.5 | 9 | 45750589 | 45751845 | processed\_transcript |
| ENSDARG00000097192 | si:ch73-59c19.2 | 16 | 46430945 | 46464147 | antisense |
